# Physical activity levels objectively measured among older adults: a population-based study in a Southern city of Brazil

**DOI:** 10.1186/s12966-017-0465-3

**Published:** 2017-02-02

**Authors:** Virgílio Viana Ramires, Fernando César Wehrmeister, Andrea Wendt Böhm, Leony Galliano, Ulf Ekelund, Soren Brage, Inácio Crochemore Mohnsam da Silva

**Affiliations:** 1 Postgraduate Program in Epidemiology, Federal University of Pelotas, Marechal Deodoro, 1160 – 3 Piso, Centro, 96020-220 Pelotas, Brazil; 2 Federal Institution for Education, Science and Technology, Camaquã Campus, Camaquã, Brazil; 3 Research Group in Accelerometry-based Physical Activity, Pelotas, Brazil; 4 Postgraduate Program in Physical Education, Federal University of Pelotas, Pelotas, Brazil; 5 Medical Research Council Epidemiology Unit, University of Cambridge, Cambridge, UK; 6 Norwegian School of Sports Science, Oslo, Norway; 7 International Center of Equity in Health, Postgraduate Program in Epidemiology, Federal University of Pelotas, Pelotas, Brazil

**Keywords:** Population-based study, Older adults, Physical activity and accelerometry

## Abstract

**Background:**

Low levels of physical activity are currently observed in all age groups around the world. Among older adults physical activity is even lower, potentially influencing quality of life, incidence of diseases and premature mortality. The aim of this study was to describe objectively measured physical activity levels among older adults residents in a Southern city of Brazil.

**Methods:**

A population-based study was carried out including people aged 60+ years living in the urban area of Pelotas. Face-to-face interviews, anthropometric measures and triaxial accelerometry (non-dominant wrist) were used to collect sociodemographic, anthropometric and physical activity, respectively. For descriptive purposes, overall physical activity was expressed as daily averages of acceleration. Time spent in light physical activity (LPA) and moderate-to-vigorous physical activity (MVPA) using different bout criteria (non-bouted, and in 1-, 5- and 10-min bouts) were calculated. Crude and adjusted analyses were performed using simple linear regression to examine the association between physical activity and exposure variables.

**Results:**

Overall, 971 individuals provided valid accelerometry data. Women spent on average more time on LPA (136.2 vs. 127.6 min per day). Men and women respectively accumulated, in average, 64.5 and 56.7 min per day of non-bouted MVPA, while these daily averages were 14.9 and 9.46 min using 5-min, and 8.1 and 4.5 min using 10-min bout MVPA. In adjusted analyses, men aged 80 years or more spent in average 45 min less LPA per day when compared to men 60-69 years and, among women, this difference was 65 min. Considering time in 5-min MVPA bouts, the youngest age group and those with a better self-perceived health accumulated more MVPA. Specifically among men, socioeconomic status was inversely associated with 5-min bout MVPA.

**Conclusion:**

The present study showed low levels of physical activity among Brazilian older adults, even lower in more advanced ages, and a different pattern for physical activity intensity between men and women.

## Background

The low levels of physical activity currently observed in all age groups around the world [1] are a concerning scenario in terms of public health, although less objective data are available in low-middle income countries. There is a large amount of evidence highlighting physical inactivity as an important risk factor for many chronic diseases [2]. Specifically in the older adults population, levels of physical activity tend to be even lower than in adults [3]. Although lower levels of physical activity influence a higher incidence of diseases and premature mortality, adequate levels of physical activity might play an important role in healthy aging [4].

Rapid demographic transitions experienced by low- and middle-income countries have been marked by the challenge regarding aging with basic quality of life [5]. Further, accurate physical activity estimates, especially in these countries, are scarce providing a limited diagnosis of physical activity levels. Due to lower complexity and relative low cost, most studies use subjective methods, such as questionnaires, to assess physical activity [6, 7]. However, among older adults population, estimates provided by these methods present lower accuracy compared to objective measurement of physical activity [8]. In general, subjective methods tend to overestimate moderate and vigorous physical activity and underestimate sedentary behavior [9, 10] and associations with health outcomes tend to be stronger using objective measures [11].

In this context, the uses of objective methods are preferable for assessing physical activity patterns among the older adults [12]. Furthermore, descriptive studies are needed to accurately measure population physical activity levels, as well as their distribution across specific population strata which could be the target of interventions. Thus, the aim of this study was to describe physical activity, measured by accelerometers in a population-based sample of older adults residents in a Southern city of Brazil, highlighting differences among sexes, nutritional status and socioeconomic status.

## Methods

### Sampling and study design

A population-based study was carried out among older adults living in the urban area of Pelotas, Rio Grande do Sul state, Brazil. Pelotas is a southern city with around 340,000 inhabitants and approximately 46,000 people aged 60 years or older [13]. Its HDI is 0.74, similar to the overall country. This study is part of a large survey which assesses general aspects of health in an older adults population. The data collection was conducted between January and August 2014 and individuals aged 60 years or older were considered older adults, according to recommendations of the World Health Organization (WHO) for low- and middle-income countries [14].

The sampling process was performed in two stages. First, a total of 488 census tracts from urban areas of Pelotas were sorted according to their average family income, based on the 2010 Demographic Census [13]. Census tracts presenting fewer than 15 older adults people were clustered. Therefore, from 469 census tracts listed, 133 were randomly selected. The second stage was the household selection within each census tract included in the survey. Based on the total number of inhabited households, a systematic selection was performed and all adults older than 60 years who were living in the selected households were invited to participate. The sampling process included 31 households per census tract and around 12 participants in each cluster were recruited.

### Data collection

A trained team carried out face-to-face interviews including questions on sociodemographic and health information. Anthropometric measures were also performed. Weight was assessed using electronic scales (Tanita^®^, model UM-080), which is able to handle up to 150 kg at a precision of 0.1 kg. For estimation of height, a knee height measurement was applied with participants seated using infants anthropometric instrument (Indaiá^®^). The final height was based on Chumlea e Guo [15] equations and this procedure is justified due to difficulties to sustain orthostatic posture in older adults population.

### Accelerometry

Following the interview, participants were invited to wear an accelerometer on their non-dominant wrist for the next 7 days, 24 h per day, including during water-based activities. The interviewers provided all important information regarding the devices and informed about a future call to schedule the accelerometer attachment. Participants wore the devices during seven consecutive days and the research team was responsible for attaching and collecting the accelerometers from the participants’ households.

The accelerometer used was the GENEActiv® (Activinsights Ltd, Kimbolton, Cambs, UK, http://www.geneactiv.org), a water-proof device which measures acceleration in three axes and provides raw data expressed in gravitational equivalent units (1000 *mg* = 1 *g*). Accelerometers were initialized to collect data in 85.7 Hz time resolution. Bed-bound and disabled older adults were considered as exclusion criteria for the accelerometer measurement.

### Accelerometer data processing

The GENEAcitiv software was used to set up and download accelerometers data. Raw data were calibrated to local gravity, scored for non-wear based on prolonged (>60 min) periods of low acceleration variability (SD < 13 mg), and abnormally high values were censored. Activity-related acceleration was calculated using the Euclidian Norm (vector magnitude of the three axes) minus 1 g (ENMO = √*x* ^2^ + y^2^ + z^2^ -1 g), and invalid data segments were imputed by the average of similar time-of-day data points from other days of the measurement (within individual). Activity intensity was estimated from 5-s aggregated time-series as average time per day spent in light, moderate and vigorous physical activities. Detailed information about these analytical procedures is available elsewhere [16–18]; these analyses were performed in R-package GGIR (http:/cran.r-project.org).

In the present study, overall physical activity is expressed by the daily average of acceleration. Light physical activity (LPA) was defined as activities representing acceleration between 50 and 99 m*g*, while activities with acceleration higher than 100 m*g* were considered as moderate to vigorous physical activity (MVPA) [16, 19]. Furthermore, specifically for MVPA, different bout criteria were adopted (non-bouted, 1-, 5- and 10 min-bout). Bouts were defined as consecutive periods in which participants spent at least 80% of this time in MVPA. LPA (non-bouted) and MVPA in 5 min-bout were the main outcomes in the association analyses. Participants providing fewer than two days of measurement were excluded from the analyses.

### Complementary variables

The following basic sociodemographic characteristics were assessed and categorized as follows: sex (men/women); age (60-69; 70-79 e ≥ 80 year); skin color (white/non-white); socioeconomic status (based on asset index and grouped as A/B – richest, C and D/E – poorest) [20]; marital status (single or married); occupational status (currently not working or currently working); self-perceived health (very good/good; regular and bad/very bad); and Body Mass Index (BMI - normal < 25 kg/m^2^; overweight > 25 and <30 kg/m^2;^ and obese > 30 kg/m^2^).

### Statistical analyses

Sample descriptive analyses are presented based on relative and absolute frequencies. ANOVA and *T*-test or Kruskal Wallis and Wilcoxon non-parametric tests were performed in order to verify the average differences among dichotomized and ordinal variables, respectively. Crude and adjusted analyses were performed using simple linear regression to verify the association between physical activity and exposure variables. All analyses were stratified by sex and took the clustering of the sample into account. Analyses were carried out in Stata (version 12.1).

The present study was submitted and approved by the Ethics Research Committee of the Medical School of the Federal University of Pelotas according to the protocol number 201324538513.1.0000.5317. The confidentiality was guaranteed for all individual information and all participants signed the research consent.

## Results

Among the 1844 older adults eligible to participate in the study, 1451 were interviewed. The losses and refusals (21.3%) were similarly distributed in terms of sexes and age groups. Overall, 971 participants (66%) had valid accelerometry data. The socioeconomic, behavioral and health characteristics from the general and analytic sample are showed in Table 1. The analytical sample included more women (62.2%), were between 60 and 69 years of age (51.1%), white (82.1%), living with a partner (56.2%), retired (79.6%), reported a socioeconomic status “C” (54.4%), overweight (42.2%), and a perception about their health classed as “good” (52.1%).

**Table 1:** Original and analytical sample characteristics according to sociodemographic and health characteristics – Pelotas/Brazil, 2014

Characteristics	Total *N* (%)	Analytical *N* (%)	*p*-value
Gender			0.700
Male	537(37.0)	367(37.8)	
Female	914(63.0)	604(62.2)	
Age			0.255
60 - 69	756(52.3)	496(51.1)	
70 - 79	460(31.8)	337(34.7)	
≥80	230(15.9)	138(14.2)	
Skin color			0.301
White	1211(83.7)	797(82.1)	
Not White	236(16.3)	174(17.9)	
Marital Status			0.090
Married	763(52.7)	546(56.2)	
Single	684(47.3)	425(43.8)	
Occupational Status			0.320
Currently not working	1084(80.4)	728(79.6)	
Currently working	264(19.6)	172(20.4)	
Socioeconomic Status			<0.001
A/B (richest)	384(27.9)	327(35.1)	
C	781(56.8)	506(54.4)	
D/E (poorest)	210(15.3)	98(10.5)	
Body Mass Índex (BMI)			0.887
Normal (BMI < 25 kg/m^2^)	385(28.2)	259(27.3)	
Overweight (BMI > 25 < 30 kg/m^2^)	571(41.9)	400(42.2)	
Obese (BMI > 30 kg/m^2^)	408(29.9)	289(30.5)	
Self-perceived health			0.885
Very good/ Good	765(53.1)	504(52.1)	
Regular	545(37.8)	375(38.7)	
Very bad/ Bad	132(9.1)	89(9.2)	

**Fig. 1:**
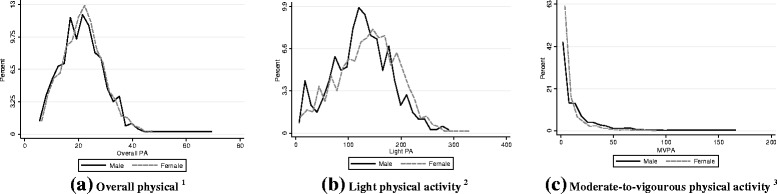
Overall physical activity (a) and average time spent per day in light (b) and moderate-to-vigorous (c) physical activity among older adults according to sex – Pelotas/Brazil, 2014. Acceleration mean per day 1. Average minutes per day spent in light physical activity non-bouted criteria. 2. Average minutes per day spent in moderate-to-vigorous physical activity (5 min bout)

The description and distribution of total, light and moderate to vigorous physical activity stratified by sex are showed in Fig. 1 and Table 2. In general, women spend more time on light intensity physical activity (136.2 vs. 127.6 min per day), while men spend more time on MVPA (15.0 vs. 8.1 min per day). Total physical activity did not differ between sexes (22.0 vs. 21.5 *mg*).

**Table 2:** Descriptive of physical activity levels among older adults – Pelotas/Brazil, 2014

	Overall PA (*mg*) Mean and 95%CI	P **	LPA (min/day) Mean and 95%CI	P **	MVPA* (min/day) Mean and 95%CI	P ^#^
Male	22.0 (21.2; 22.9)	0.307	128 (122; 133)	0.024	15 (13; 17)	<0.001
Female	21.5 (20.9; 22.1)		136 (132; 140)		8 (7; 9)	
Total	21.7 (21.2; 22.2)		133 (129; 136)		11 (10; 12)	

*MVPA* moderate to vigorous physical activity, *LPA* light physical activity

*MVPA 5 min bout

** *T* test

^#^ Wilcoxon rank-sum test

**Fig. 2:**
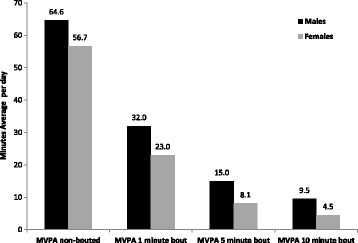
Average minutes per day spent in MVPA according to different bout criteria among older adults according to sex– Pelotas/Brazil, 2014

Important differences were observed in MVPA in accordance to the different bout criteria (Fig. 2). When no bout criterion was used, men and women performed an average of 64.5 and 56.7 min per day of MVPA, respectively. However, when the 1-min bout criterion was considered, this estimate decreased by approximately 50%. Finally, when the 5- and 10-min MVPA bouts were evaluated, the average daily time spent in these intensities were 14.9 and 9.5 min per day among men and 8.1 and 4.5 min per day among women, respectively.

Table 3 shows the association between socioeconomic status, behavioral and health characteristics with light intensity physical activity. Men aged 80 years or more spent on average 45 min less in LPA per day when compared to the 60 – 69 years-old age group. Among women, this difference was 65 min. Furthermore, minutes spent in LPA were slightly lower (approximately 10 min) among obese women and those single, compared to their counterparts. Older adults who reported being employed performed on average 20 min per day more of LPA than those who did not work. Men and women who considered their health bad or very bad accumulated in average 46 and 33 min less time per day spent in light intensity physical activity than those who considered their health very good or good, respectively. There was no difference between LPA and nutritional status and marital status among men; and between LPA and socioeconomic status among both men and women.

**Table 3:** Crude and adjusted association between light intensity physical activity with sociodemographic and health characteristics in older adults – Pelotas/Brazil, 2014

Variable	Male						Female					
	*N*	Crude (Mean and IC95%)	*p*	*n*	**Adjusted (Mean and IC95%)	*p*	*n*	Crude (Mean and IC95%)	*p*	*n*	**Adjusted (Mean and IC95%)	*p*
Age			<0.001			<0.001			<0.001			<0.001
60 - 69	188	144 (137; 151)		173	143 (136; 150)		308	156 (151; 161)		270	151 (146; 157)	
70 - 79	128	117 (108; 127)		117	122 (112; 132)		209	130 (122; 137)		176	134 (126; 143)	
≥80	51	93 (77; 109)		48	98 (81; 115)		87	83 (72; 93)		69	86 (74; 99)	
Socioeconomic status			0.62			0.5			0.2			0.8
A/B (richest)	136	132 (123; 142)		131	129 (120; 138)		191	140 (132; 147)		173	136 (129; 144)	
C	180	125 (118; 132)		172	127 (120; 134)		326	136 (130; 142)		288	138 (132; 144)	
D/E (poorest)	36	127 (108; 146)		35	140 (120; 159)		62	124 (108; 140)		54	132 (117; 148)	
Body Mass Índex (BMI)			0.2			0.3			0.3			0.01
Normal (BMI < 25 kg/m^2^)	112	122 (112; 132)		107	127 (117; 137)		147	136 (125; 146)		126	141 (131; 151)	
Overweight (BMI > 25 < 30 kg/m^2^)	161	135 (127; 143)		154	133 (125; 141)		239	141 (133; 148)		200	142 (134; 149)	
Obese (BMI > 30 kg/m^2^)	83	126 (116; 137)		77	124 (115; 133)		206	132 (125; 139)		189	129 (123; 135)	
Marital status			0.05			0.09			<0.001			0.04
Single	78	117 (105; 129)		71	119 (108; 131)		347	127 (121; 133)		296	133 (128; 138)	
Married	289	130 (124; 137)		267	132 (125; 138)		257	149 (142; 155)		219	142 (135; 149)	
Occupational status			<0.001			0.001			<0.001			<0.001
Currently not working	262	118 (111; 124)		239	123 (117; 129)		466	130 (125; 135)		437	133 (128; 138)	
Currently working	110	153 (143; 162)		99	144 (133; 155)		84	170 (159; 181)		78	158 (147; 169)	
Self-perceived health			0.003			0.001			<0.001			<0.001
Very good/ Good	200	135 (128; 143)		186	134 (127; 141)		304	147 (142; 153)		260	144 (138; 149)	
Regular	146	123 (114; 131)		135	127 (118; 136)		229	129 (122; 137)		199	135 (127; 143)	
Very bad/Bad	20	89 (69; 110)		17	91 (70; 113)		69	108 (92; 124)		56	111 (96; 127)	

* Non-parametric Kruskall Wallis test for ordinal variables and Wilcoxon test for dichotomized variables

** Adjusted for: age, socioeconomic status, BMI, marital status, occupational status and self-perceived health

Table 4 shows the results from the association between MVPA and socio-demographic and health variables considering the 5- min bout criterion. Men and women aged 80 years or older performed on average 16 and 10 min less MVPA per day, respectively than the 60-69 years-old group. Men from less privileged social status were more active in MVPA in relation to the ones from higher social status (14 min on average), as well as those of normal weight in relation to those categorized as obese (8 min). Older adults with a very good or good self-reported health perception spent more time on MVPA than those who considered their health bad or very bad (16 min per day among men and 7 min per day among women).

**Table 4:** Crude and adjusted association between MVPA (5-min bout) and sociodemographic and health characteristics in older adults – Pelotas/Brazil, 2014

Variable	Male						Female					
	*n*	Crude (Mean and IC95%)	*p*	*n*	Adjusted (Mean and IC95%)	*p*	*n*	Crude (Mean and IC95%)	*p*	*n*	Adjusted (Mean and IC95%)	*p*
Age			<0.001			<0.001			<0.001			<0.001
60 - 69	188	20 (16; 23)		173	20 (17; 23)		308	12 (10; 13)		270	11 (10; 13)	
70 - 79	128	13 (10; 16)		117	13 (10; 17)		209	6 (4; 7)		176	6 (5; 8)	
≥80	51	3 (2; 4)		48	4 (1; 6)		87	1 (0; 1)		69	1 (0; 2)	
Socioeconomic status			0.03			0.001			0.2			0.4
A/B (richest)	136	12 (10; 15)		131	12 (9; 14)		191	9 (7; 11)		173	8 (6; 9)	
C	180	15 (12; 19)		172	16 (13; 20)		326	8 (6; 9)		288	8 (7; 9)	
D/E (poorest)	36	24 (13; 32)		35	26 (19; 34)		62	8 (4; 12)		54	11 (7; 15)	
Body Mass Índex (BMI)			0.002			0.002			0.3			0.2
Normal (BMI < 25 kg/m^2^)	112	18 (13; 22)		107	18 (14; 23)		147	8 (6; 10)		126	9 (7; 10)	
Overweight (BMI > 25 < 30 kg/m^2^)	161	17 (13; 20)		154	16 (13; 20)		239	9 (7; 11)		200	9 (8; 11)	
Obese (BMI > 30 kg/m^2^)	83	10 (6; 13)		77	10 (7; 13)		206	8 (6; 10)		189	7 (5; 9)	
Marital status			0.4			0.9			<0.001			0.3
Single	78	16 (10; 22)		71	15 (10; 21)		347	7 (5; 8)		296	8 (6; 9)	
Married	289	15 (13; 17)		267	15 (13; 18)		257	10 (8; 12)		219	9 (7; 11)	
Occupational status			0.001			0.04			<0.001			0.9
Currently not working	262	12 (10; 14)		239	14 (12; 16)		466	8 (6; 9)		437	8 (7; 9)	
Currently working	110	21 (16; 27)		99	19 (14; 24)		84	12 (8; 15)		78	8 (5; 12)	
Self-perceived health			<0.001			<0.001			<0.001			<0.001
Very good/ Good	200	18 (15; 21)		186	19 (15; 22)		304	10 (9; 12)		260	10 (8; 12)	
Regular	146	12 (9; 16)		135	13 (9; 16)		229	7 (5; 8)		199	7 (6; 9)	
Very bad/Bad	20	4 (2; 7)		17	3 (0; 7)		69	3 (2; 5)		56	3 (2; 5)	

* Non-parametric Kruskall Wallis test for ordinal variables and Wilcoxon test for dichotomized variables

# MVPA = Moderate to vigorous intensity physical activity

**Adjusted for: age, socioeconomic status, BMI, marital status, occupational status and self-perceived health

## Discussion

The present study described levels of physical activity objectively measured among older adults in a population-based sample from Brazil, providing relevant evidences from a middle-income country which are still scarce in the literature. The average time spent per day in MVPA was relatively low among older adults and varied according to different analytical procedures. Moreover, important differences were found in intensities of physical activity according to sexes. Women spent more time in LPA, while men accumulated more time of MVPA, similarly to studies from high-income settings [3, 21]. The oldest participants, those currently not working (retired or unemployed) and reporting a poor self-perceived health presented lower levels of light and moderate to vigorous intensity physical activity.

The use of raw accelerometry presents many advances, such as transparency in the analytical process and enhanced comparability between data collected from different devices; however, there are still only limited triaxial wrist acceleration data to compare current results to, owing to this attachment site only becoming more commonly used in very recent studies [12, 22]. Among older adults, a study with similar methodology was an English occupational cohort study [11] aimed to compare effects of physical activity on adiposity, measured by accelerometers and questionnaire. In this study, the average daily acceleration, estimate of global physical activity (total volume), without intensities thresholds, was 23.4 mg among men and 23.1 mg among women. These results are similar to our findings in terms of daily volume of physical activity and the absence of differences between men and women. Further comparisons also might be carried out with data from a methodological study, which provided descriptive data from three studies from different countries among adults. The average daily acceleration in United Kingdom (mean of age: 50.3 years), Kuwait (mean of age: 43.0 years) and Cameroon (mean of age: 40.3 years) were 31.8 mg, 24.6 mg and 34.5 mg, respectively.

It is also important to highlight effect of applying different bout criteria to data summarize in 5 s epoch. Time spent in MVPA decreased about 45%, 19% and 11% when 1, 5 and 10-min bout were used, respectively. This observation corroborates prior findings in adult populations [16, 23] suggesting that the use of different bout criteria considerably affects the final estimate of MVPA. These methodological differences might be even more pronounced among older adults compared to other population groups, especially since older adults are less likely to sustain MVPA for longer periods [24]. Ortileb et al. (2014), for example, found that 47.6% of older adults participants did not reach at least one 10-min bout of MVPA daily [25]. When applying the WHO physical activity recommendations for public health [26], 35.7% and 11.9% of the participants achieved guidelines when no bout and 10-min bout criteria were applied, respectively.

In addition, intermittent exercises, which do not necessarily reach the bout criteria, may also be important to improve health and quality of life among older adults, as improvements in locomotor and neuromuscular performance [27], aerobic capacity [28], muscular strength and blood pressure [29] have been demonstrated. Therefore, objective methods to assess physical activity should be taken into account bout criteria for this population group, especially when considering that older adults tend to perform shorter duration exercises [9].

Previous accelerometer-based studies suggest a decrease in physical activity by increasing age [3, 21, 30, 31]. Although all reasons are not exactly identified (30), it might be due to difficulty in mobility, general health status and self-efficacy. Moreover, retirement may decrease transport related physical activity and work related physical activity, which also might be replaced by leisure-time activity [32]. Increase in physical activity between older adults is a relevant factor for improvements on quality of life, especially considering the higher risk of morbidities attributed to aging that can be prevented through an active life style [25].

Despite the existing evidence in the literature considering the wider opportunities and knowledge about physical activity and its relevance, our results showed that those of lower SES were more active in MVPA as compared to higher SES groups. This may partly be explained by differences in transport related physical activity. A recent study from Mexican found an inverse association between active life style and car use, and that lower SES groups walk, cycle or use public transport when commuting [32]. Another study suggested that lower socioeconomic groups were less active in MVPA which was closely related to the characteristics of their place of residence [33]. Thus, studies examining associations between physical activity and socioeconomic status should consider both the physical activity domains assessed as well as the locations where activity take place. Studies addressing specifically leisure-time activities tend to identify positive associations. On the other hand, studies evaluating overall physical activity, based on accelerometry for example, tend to verify higher heterogeneity and their results, varying according to different settings in which the research was carried out.

The present results should be interpreted considering the following limitations. A third of the participants were not given the opportunity to wear accelerometers due to the limited number of available devices during the period of contact with the older adults population. However, the analytical sample is likely representative of the city of Pelotas, as the missingness was randomly distributed, except for the richest group which is slightly over representated. Furthermore, the cross-sectional nature of the present study preclude causal inference and the association (or lack of association), between physical activity and self-perceived health and BMI might be due to reverse causality.

## Conclusions

The rapid demographic transition which results in population aging, especially in low- and middle-income countries, is characterized by several public health challenges. Participation in physical activity among the older adults is currently an important strategy to prevent chronic diseases and to promote health and quality of life. In this regard, it is relevant to describe physical activity levels measured as accurate as possible, as well as identify specific groups which should be targeted by public health policies. Our results suggest low levels of physical activity in a population-based sample of Brazilian older adults, a substantial reduction in activity lower by more advanced ages, higher levels of activity in lower SES groups and a different pattern of physical activity intensities between men and women.
